# Summer Diarrhœa

**Published:** 1889-09

**Authors:** 


### SUMMER DIARRHŒA.

Many interesting articles on this much-discussed subject have been contributed to medical literature during the present year. Many investigators have been diligently searching for the pathogenic micro-organisms without very definite results. Many have suggested modifications of treatment, though few radical changes have been advocated. Antiseptics, from which so much was expected a year ago, have not yielded satisfactory results. They destroy the normal, conservative bacteria as well as the pathogenic, are unreliable, and where there is inflammation which may be, and usually is, present in some part of the intestinal tract, they are undoubtedly harmful. In the simple fermentative diarrhœas they are valuable. It is a well established fact that during infancy the contents of the whole alimentary tract are slightly acid. Pfieffer, contrary to the generally accepted theory that the greenish stools are due to excessive acidity of the intestinal contents, believes they are the result of alkalinity, and carefully conducted experiments seem to bear him out. Lactic and nitro-hydro-chloric acids, especially the former, are very highly lauded by a number of eminent writers. Lactic acid is generally directed in 2 per cent. aqueous solution, (9½ grs. to the ounce of water,) to which a little syrup is added, and of which half a drachm is given fifteen or twenty minutes after each nursing or feeding.

Ehring's method of washing out the stomach and intestines has proven to be, in competent hands, a very valuable addition to the therapeutics of this troublesome affection. For washing out the stomach he uses a Nelaton catheter No. 8–10, to which is attached a Y shaped glass tube, having two rubber tubes attached to the other extremities.

Luke-warm water is poured into the upper tube until the stomach is full, then it is compressed and the fluid is allowed to run out through the lower tube, pressure being made at the same time over the region of the stomach. This is repeated until the water comes away perfectly clear. The method is especially valuable in persistent vomiting, and gastric catarrh. Intestinal irrigation produces excellent results in enterocolitis. The agents generally preferred are luke-warm water, or a solution of common salt, three grains to the ounce of water, injected through a rectal tube introduced high up into the intestine.

Copious injections should be used, after which astringents may be employed if indicated.

Calomel still holds its place as one of our most valuable therapeutic agents.

Bismuth also continues, as it justly deserves to do, a favorite remedy.

Diet is, of course, all important. Where there is obstinate vomiting, all food should be withheld from four to six hours, so as to give the stomach complete rest; then milk should be substituted by barley-water with white of an egg, meat broths, etc., given in very small quantities and gradually increased as the stomach becomes more tolerant; after which sterilized milk should be returned to very gradually.

Stimulants are almost always indicated and usually well borne. Fresh air sterilization of the milk by Sohxlet's method, with perfect cleanliness and greatest care in the preparation of the diet, are the sheet anchors for the prevention of the disease in hand-fed children. The stools should be disinfected, especially in large cities.

